# A case of metastatic cancer with markedly elevated PSA level that was not detected by repeat prostate biopsy

**DOI:** 10.1186/1756-0500-7-64

**Published:** 2014-01-29

**Authors:** Hiromichi Iwamura, Shingo Hatakeyama, Yoshimi Tanaka, Toshikazu Tanaka, Noriko Tokui, Hayato Yamamoto, Atsushi Imai, Takahiro Yoneyama, Yasuhiro Hashimoto, Takuya Koie, Kazuaki Yoshikawa, Chikara Ohyama

**Affiliations:** 1 Department of Urology, Hirosaki University Graduate School of Medicine, 5 Zaifu-cho, Hirosaki 036-8562, Japan; 2 Department of Urology, Mutsu General Hospital, 035-0071 Mutsu, Japan

**Keywords:** Metastatic prostate cancer, Negative prostate biopsy, Elevated PSA level

## Abstract

**Background:**

Prostate-specific antigen (PSA) is a widely used specific tumor marker for prostate cancer. We experienced a case of metastatic prostate cancer that was difficult to detect by repeat prostate biopsy despite a markedly elevated serum PSA level.

**Case presentation:**

A 64-year-old man was referred to our hospital with lumbar back pain and an elevated serum PSA level of 2036 ng/mL. Computed tomography, bone scintigraphy, and magnetic resonance imaging showed systemic lymph node and osteoblastic bone metastases. Digital rectal examination revealed a small, soft prostate without nodules. Ten-core transrectal prostate biopsy yielded negative results. Androgen deprivation therapy (ADT) was started because of the patient’s severe symptoms. Twelve-core repeat transrectal prostate biopsy performed 2 months later, and transurethral resection biopsy performed 5 months later, both yielded negative results. The patient refused further cancer screening because ADT effectively relieved his symptoms. His PSA level initially decreased to 4.8 ng/mL, but he developed castration-resistant prostate cancer 7 months after starting ADT. He died 21 months after the initial prostate biopsy from disseminated intravascular coagulation.

**Conclusion:**

CUP remains a considerable challenge in clinical oncology. Biopsies of metastatic lesions and multimodal approaches were helpful in this case.

## Background

The serum prostate-specific antigen (PSA) level is widely used for prostate cancer screening [1]. As the PSA level may also be elevated in patients with prostatic inflammation and benign prostatic hypertrophy, definitive diagnosis of prostate cancer requires prostate biopsy. However, prostate cancer is occasionally difficult to diagnose by prostate biopsy, even in patients with markedly elevated PSA levels. We report a case of metastatic prostate cancer in a patient who underwent three biopsy procedures that all yielded negative results.

## Case presentation

**Figure 1:**
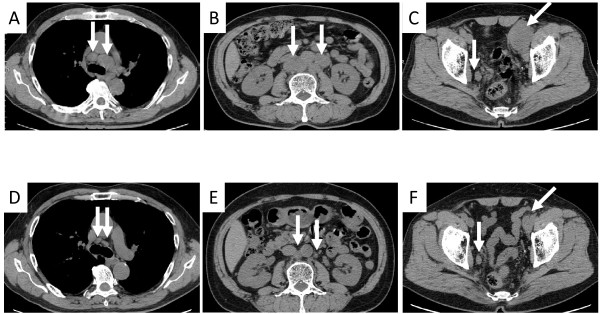
**Computed tomography before and after treatment.** Pre-treatment images showed enlarged mediastinal (**A**, arrows), para-aortic (**B**, arrows), and iliac (**C**, arrows) lymph nodes. Images nine months after ADT showed a partial response of the mediastinal (**D**, arrows), para-aortic (**E**, arrows), and iliac (**F**, arrows) lymph nodes.

**Figure 2:**
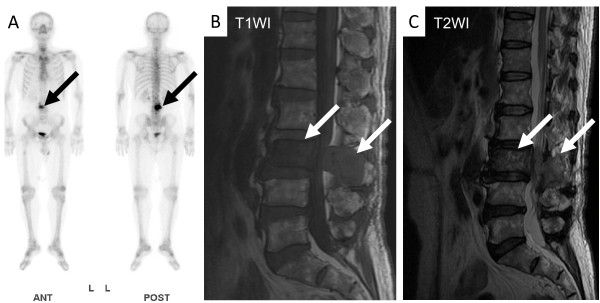
Bone scintigraphy (A) and magnetic resonance imaging (B, C) showed osteoblastic lumbar spine metastases (arrows).

**Figure 3:**
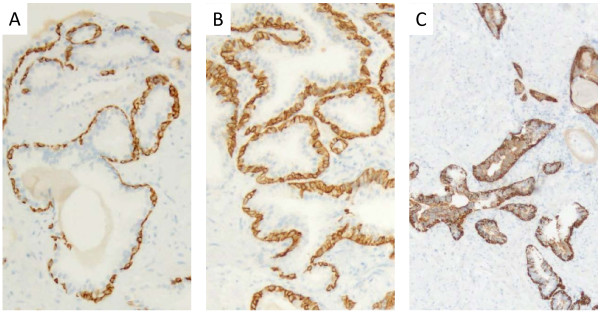
**Histopathological examination findings.** The first 10-core **(A)** and second 12-core **(B)** transrectal prostate biopsies and the transurethral resection biopsy (5 g) **(C)** showed benign prostatic hyperplasia. The estimated total weight of the prostate at the time of transurethral resection was 16 g. The 34βE12 anti-cytokeratin monoclonal antibody was positive in all specimens. Original magnification: ×40.

A 64-year-old man was referred to our hospital with lumbar back pain and an elevated serum PSA level of 2036 ng/mL. Computed tomography showed enlarged mediastinal, para-aortic, and iliac lymph nodes (Figure 1A,B,C). Bone scintigraphy and magnetic resonance imaging showed osteoblastic lumber spine metastases (Figure 2). Digital rectal examination revealed a small, soft prostate without nodules. The estimated total weight of the prostate was 34 g. Because of the markedly elevated PSA level, we considered that biopsies of the metastases were not essential. Ten-core transrectal prostate biopsy yielded negative results (Figure 3A). Because the patient was experiencing severe fatigue and pain, we regarded treatment to be a higher priority than histological diagnosis. We diagnosed TxN1M1b prostate cancer based on the clinical findings, and started androgen deprivation therapy (ADT) with a luteinizing hormone-releasing hormone agonist and an anti-androgen agent (bicalutamide), together with zoledronic acid therapy. To obtain a definitive diagnosis, 12-core repeat prostate biopsy was performed 2 months later and transurethral resection biopsy was performed 5 months later. The resected transurethral specimen weighed 5 g (the estimated total weight of the prostate: 16 g), but did not contain prostate cancer tissue (Figure 3B,C). The patient refused further prostate cancer screening because ADT effectively relieved his symptoms. Nine months after the initial prostate biopsy, his enlarged lymph nodes had shrunk in size (Figure 1D,E,F) and his PSA level had decreased to 4.8 ng/mL. However, he did not attend his routine follow-up appointments and was noncompliant with ADT, and developed castration-resistant prostate cancer 7 months after starting ADT. We administered five courses of docetaxel-based chemotherapy, but his response was inadequate. The patient died 21 months after the initial prostate biopsy from disseminated intravascular coagulation. His family refused to allow an autopsy.

## Discussion

Carcinoma of unknown primary (CUP) is an unusual malignant condition in which metastases are documented without the identification of the primary site, even after intensive screening [2]. CUP accounts for 3–5% of all cancers [3]. Extensive work-up with specific pathology investigations (immunohistochemistry, electron microscopy, molecular diagnosis) and modern imaging technology (computed tomography [CT], magnetic resonance imaging [MRI], mammography, positron emission tomography [PET] scan) have resulted in some improvements in diagnosis; however, the primary site remains unknown in most patients, even after autopsy. Therefore, it remains a major problem in clinical oncology. CUP is considered the seventh or eighth most common type of malignancy and fourth most common cause of cancer-related death [4]. In a meta-analysis of 12 postmortem studies, the primary tumor was identified in 644 (73%) of 884 patients. The most common primary sites were the lung (27%), pancreas (24%), liver or bile duct (8%), kidney or adrenal gland (8%), colon or rectum (7%), genital tract (7%), and stomach (6%) [5]. In patients with CUP with skeletal metastases, the prostate was the second most common primary site. A recent review of CUP in the urogenital system reported the frequency of CUP of the prostate to be 4% [6].

The reason most such primary tumors cannot be detected is unclear. Common hypotheses include spontaneous regression or immune-mediated destruction of the primary tumor, and inherently small primary tumors [2,4,7,8]. Therefore, the utility of traditional CT and MRI in detecting small lesions and non-enhancing lesions in normal-sized structures is limited. Relatively recent studies showed the clinical value of ^18^ F-fluoro-2-deoxyglucose positron emission tomography/computed tomography (^18^ F-FDG PET-CT) in detecting primary tumors in patients with CUP [7,9]. Han et al. [9] reported that ^18^ F-FDG PET-CT whole-body imaging correctly detected primary tumors in 54 of 120 patients (42.5%). The sensitivity, specificity and accuracy of ^18^ F-FDG PET-CT in detecting primary tumor sites were 91.5%, 85.2% and 88.3%, respectively.

Two opposite approaches have been adopted in CUP diagnostic procedures: one strategy is a “shotgun modality,” consisting of multiple examinations aimed at identifying the primary tumor; the other is a “nihilistic modality,” which adopts palliative therapy for the metastatic disease. Because overall survival is poor in patients with CUP (median: 6–14 months) [10], a reasonable intermediate diagnostic strategy consists of undertaking procedures with specific targets and low cost/benefit ratios [6].

In the present case, according to the guidelines for the management of CUP [3], osteoblastic bone metastases in a patient with a markedly elevated PSA level suggests metastatic PSA^+^ cancer, and hormone-based treatment is recommended if the primary lesion is not detected within 1 month. As a PSA level > 2000 ng/mL strongly suggests prostate cancer, we started ADT before obtaining a histological diagnosis. As expected, ADT was effective in improving the patient’s symptoms. Although we tried to obtain a definitive diagnosis of prostate cancer by repeat prostate biopsies including transurethral resection biopsy, all biopsies yielded negative results. Because initial biopsies only detect 65–77% of prostate cancers, repeat biopsies are frequently performed [11,12]. Djavan et al. reported prostate cancer detection rates for biopsies 1, 2, 3 and 4 to be 22%, 10%, 5% and 4%, respectively [11]. Roehl et al. reported prostate cancer detection rates from biopsies were 29%, 17%, 14%, 11%, 9% and 7%, for biopsy procedures 1–6, respectively [12]. These reports indicate that nearly a quarter of prostate cancers eventually detected in these screening studies were missed by the initial biopsy, and four prostate biopsies are needed to detect 99% of prostate cancers. Therefore, repeat biopsies are common for suspected prostate cancer. However, in the present case, the repeat biopsies may not have markedly increased the likelihood of definitive diagnosis because the number of biopsy cores was small, the same transrectal approach was used after the initial negative biopsy, and transurethral resection biopsy after ADT had low possibility of total resection of the tissue including cancer. Biopsies of the periprostatic area or via the perineal approach before ADT may have yielded a definitive diagnosis.

Only four cases of adenocarcinoma in ectopic prostate tissue have been reported [13-16]. Over 80% of ectopic prostate tissue is located in the prostatic urethra, and the second most common site is the neck of the urinary bladder [17]. In the present case, we visualized the entire urethra at the time of transurethral resection biopsy, but did not detect any ectopic nodules. The likelihood of metastasis from cancer in ectopic prostate tissue seems low.

To our knowledge, only nine cases of prostate cancer that could not be detected by repeat prostate biopsy have been reported (Table 1) [18-24]. Digital rectal examination revealed a small, soft prostate in all cases. Seven of the cases (88%) were diagnosed by biopsy of metastatic lesions, primarily in the bones or lymph nodes. Definitive diagnosis in such cases may depend on biopsy of a metastatic lesion. However, according to various reports [2,4,6,25,26], the primary site becomes obvious in only 15–20% of live patients, and 15–25% remain undefined even at postmortem examinations. Thus, biopsies of metastatic sites may be helpful, but do not always locate primary tumors [27].

**Table 1:** Reported patients with a high PSA level and prostate cancer not diagnosed by prostate biopsy

	**Authors**	**Age**	**PSA**	**Prostate biopsy (core numbers)**				**Biopsy of metastatic site**
				**1st**	**2nd**	**3rd**	**4th**	
1	Sato, *et al.*	73	100	Transrectal (6)				Cervical lymph node
2	Nakata, *et al.*	61	56.3	Transrectal (9)	Transrectal (2)	Perineal (10)		Pubic bone
3	Ueda, *et al.*	80	259	Perineal (6)	Perineal (12)			Pubic bone
4	Wakatabe, *et al.*	80	400	Transrectal (8)	Perineal (22)			Not performed
5	Wakatabe, *et al.*	69	96	Transrectal (4)	Perineal (22)			Bone
6	Makino, *et al.*	75	4222	Perineal (12)	Perineal (14)	Perineal (15)		Iliac bone
7	Shin*, et al.*	75	439	Transrectal (20)	TURP			Ureter
8	Fukumoto, *et al.*	66	88.1	Perineal (8)	Perineal (10)	Perineal (17)	Transrectal (3)	External iliac lymph nodes
9	Present case	64	2036	Transrectal (10)	Transrectal (12)	TURP		Not performed

TURP: transurethral resection of the prostate.

Although the serum PSA level is widely used as a marker for prostate cancer, PSA may also be expressed by cancers of the colon, liver, pancreas, kidney, adrenal gland, skin, mammary gland, ovary, and salivary glands [28,29]. When repeat prostate biopsies are negative, the possibility of other malignancies should be considered. Multimodal approaches sometimes detect primary malignancies that are potentially responsive to treatment. Nevertheless, CUP remains a diagnostic and therapeutic challenge for both patients and physicians in spite of recent laboratory and imaging advantages.

## Conclusion

CUP remains a considerable challenge in clinical oncology. Biopsies of metastatic lesions and multimodal approaches that included ^18^ F-FDG PET-CT were helpful in this case.

## Consent

Written informed consent was obtained from the patient’s family for publication of this case report and any accompanying images. A copy of the written consent is available for review by the Editor-in-Chief of this journal.

## Abbreviations

ADT: Androgen deprivation therapy; CUP: Carcinoma of unknown primary; PSA: Prostate-specific antigen.

## Competing interests

The authors declare that they have no competing interests.

## Authors’ contributions

HI drafted the manuscript. SH participated in drafting of the manuscript. YT, TT, NT, HY, AI, TY, YH, TK and KY performed clinical follow-up and contributed to drafting of the manuscript. CO was responsible for the concept, design, interpretation of data, and critical revision of the manuscript. All authors read and approved the final manuscript.

## Authors’ information

H.I.: postgraduate student, physician; S.H.: lecturer; Y.T.: postgraduate student, physician; T.T.: postgraduate student, physician; N.T.: postgraduate student, physician; H.Y.: assistant professor; A.I.: assistant professor; T.Y.: lecturer; Y.H.: associate professor; T.K.: associate professor; K.Y.: attending doctor and head of the Department of Urology, Mutsu General Hospital; C.O.: professor and chairman of the Department of Urology, Hirosaki University Graduate School of Medicine.
